# Effect of endoscopic resection on short-term surgical outcomes of subsequent laparoscopic gastrectomy: a meta-analysis

**DOI:** 10.1186/s12957-021-02230-5

**Published:** 2021-04-14

**Authors:** Dong Peng, Yu-Xi Cheng, Gang Liao

**Affiliations:** Department of Gastrointestinal Surgery, The First Affiliated Hospital of Chongqing Medical University, Chongqing, 400016 China

**Keywords:** Endoscopic resection, Laparoscopic gastrectomy, Gastric cancer, Meta-analysis

## Abstract

**Background:**

Endoscopic resection (ER) might affect subsequent laparoscopic gastrectomy (LG) because of the electrical coagulation, but the effect remains controversial. The purpose of this meta-analysis was to analyze the effect of ER on the short-term surgical outcomes of subsequent LG.

**Materials and methods:**

The PubMed, EMBASE, and Cochrane Library databases were searched to find eligible studies published from inception to March 21, 2021. Short-term surgical outcomes were compared between the ER-LG group and the LG-only group. The registration ID of this current meta-analysis on PROSPERO is CRD42021238031.

**Results:**

Nine studies involving 3611 patients were included in this meta-analysis. The LG-only group had a higher T stage (T1-T2: OR=2.42, 95% CI=1.09 to 5.34, *P*=0.03; T3-T4: OR=0.41, 95% CI=0.19 to 0.91, *P*=0.03) than the ER-LG group. The ER-LG group showed a shorter operation time than the LG-only group (MD=−5.98, 95% CI=−10.99 to −0.97, *P*=0.02). However, no difference was found in operation time after subgroup analysis of propensity score matching studies. No significant difference was found in intraoperative blood loss, time to first oral feeding, or postoperative hospital stay between the ER-LG group and the LG-only group. And no significance was found in overall complications (OR=1.16, 95% CI=0.89 to 1.50, *P*=0.27), complications of grade ≥ II (OR=1.11, 95% CI=0.71 to 1.73, *P*=0.64), complications of grade ≥ III b (OR=1.47, 95% CI=0.49 to 4.43, *P*=0.49) between the ER-LG group and the LG-only group.

**Conclusions:**

ER did not affect subsequent LG in terms of short-term outcomes, and the ER-LG group might have a shorter operation time than the LG-only group.

## Introduction

Gastric cancer is one of the most common gastrointestinal tumors worldwide [1, 2]. Early gastric cancer is defined as tumors confined to the mucosa or submucosa, and some of them can be cured by endoscopic resection (ER) [3, 4]. ER, including endoscopic submucosal dissection (ESD) and endoscopic mucosal resection (EMR), may be another option for the treatment of early gastric cancer without lymph node metastasis due to its quick recovery time and low cost [5, 6]. However, ER-related electrocoagulation can cause large iatrogenic ulcers, which will take 4-8 weeks to completely heal. Moreover, ER may cause edema, fibrosis, and even adhesions of the stomach and the surrounding tissues, which may increase the surgical difficulty of subsequent gastrectomy [7].

Open gastrectomy has been the main method of gastric cancer for a long time. It was not until 1994 that Kitano first described the efficacy of laparoscopic gastrectomy (LG) in early gastric cancer [8]. Furthermore, LG has had rapid development and popularity due to the minimal invasion, less blood loss, less need for painkillers, and a faster recovery over the past few decades [9]. LG is an acceptable treatment option for gastric cancer, and has better short-term effects and similar long-term oncological effects, especially for early gastric cancer [10–12].

ER might affect subsequent LG because of the electrical coagulation, and previous studies on its effect on LG remain controversial [13–16]. The main purpose of this meta-analysis was to analyze the effect of ER on short-term surgical outcomes after subsequent LG, including postoperative hospital stay, time to first oral feeding, complications, intraoperative blood loss, retrieved lymph nodes, and metastatic lymph nodes of subsequent LG.

## Materials and methods

The current meta-analysis followed the Preferred Reporting Items for Systematic Reviews and Meta-Analyses (PRISMA) statement [17]. The registration ID of this current meta-analysis on PROSPERO is CRD42021238031, and the link is https://www.crd.york.ac.uk/prospero/display_record.php?ID=CRD42021238031.

### Literature search

PubMed, EMBASE, and the Cochrane Library database were searched by two authors independently through March 21, 2021. The search strategy was performed with the following items: (“endoscopic resection” OR “endoscopic submucosal dissection” OR “endoscopic mucosal resection”) AND (“laparoscopic gastrectomy” OR “laparoscopy-assisted gastrectomy”) AND (“gastric cancer” OR “gastric carcinoma” OR “gastric neoplasms” OR “stomach cancer” OR “stomach carcinoma” OR “stomach neoplasms”). The publication language had no limitations in this search.

### Inclusion and exclusion criteria

The inclusion criteria for this meta-analysis were as follows: 1, studies comparing the effect of the ER-LG group and LG-only group on laparoscopic surgery for gastric cancer; and 2, studies reporting at least one short-term outcome: intraoperative blood loss, operative time, total number of retrieved lymph nodes, the metastatic lymph nodes, postoperative complications, and postoperative hospital stay. The exclusion criteria were as follows: 1, studies were case reports, reviews, letters, conferences, or comments; and 2, publications with insufficient data that could not be extracted. For studies with overlapping patient groups, the most recent or those with larger sample sizes were included. All disagreements about inclusion were solved by group discussion.

### Study selection

Two authors searched the databases independently. The title and abstract of the articles were screened for relevance first, and the full texts were then evaluated according to the inclusion and exclusion criteria. All disputes were resolved through internal discussion, and if the two authors had disagreed, the third author made a final judgment.

### Data extraction

The data from the included literature were extracted by two authors respectively. The contents extracted were as follows: first author, publication year, study date, country, baseline information, surgical methods, reconstruction methods, intraoperative blood loss, operation time, total number of retrieved lymph nodes, the metastatic lymph nodes, conversion to open surgery, postoperative complications, and postoperative hospital stay. When the two authors could not reach an agreement, it was determined by a third author.

### Postoperative complications

The severity of postoperative complications in this meta-analysis was performed by the Clavien-Dindo classification [18]. According to this classification, grade ≥ II required at least a pharmacological treatment with drugs and grade ≥ III b requires at least a surgical or endoscopic intervention under general anesthesia [18]. Beyond that, the specific complications including anastomotic leakage, anastomotic stenosis, intestinal obstruction, abdominal infection, gastric stasis, wound infection, pancreatic leakage, post-operative bleeding, and short-term death were extracted as well.

### Outcomes

The primary outcome of the current meta-analysis was the postoperative complications. Secondary outcomes included intraoperative blood loss, operation time, total number of retrieved lymph nodes, the metastatic lymph nodes, time to first oral feeding, conversion to open surgery, and postoperative hospital stay.

### Quality assessment

The Newcastle-Ottawa Scale (NOS) was used to evaluate the quality of the included studies. The nonrandomized studies were judged from three perspectives: selection of study comparisons, comparability between groups, and the determination of results [19].

### Statistical analysis

In the meta-analysis, continuous variables are presented as the mean and standard deviation (SD), and dichotomous variables are presented as proportions. For dichotomous and continuous variables, odds ratios (ORs) and mean differences (MDs) were calculated, respectively, and 95% confidence intervals (CI) were calculated. The value of *I*^2^ and the results of the chi-squared test were used to assess the statistical heterogeneity [20, 21]. High heterogeneity was considered when *I*^2^ > 50%, the random effects model was used, and *P*<0.1 was considered statistically significant. The fixed effects model was used for the studies with *I*^2^≤50%, and *P*<0.05 was considered statistically significant. This meta-analysis was performed with RevMan 5.3 (The Cochrane Collaboration, London, UK).

## Results

### Study selection

**Fig. 1:**
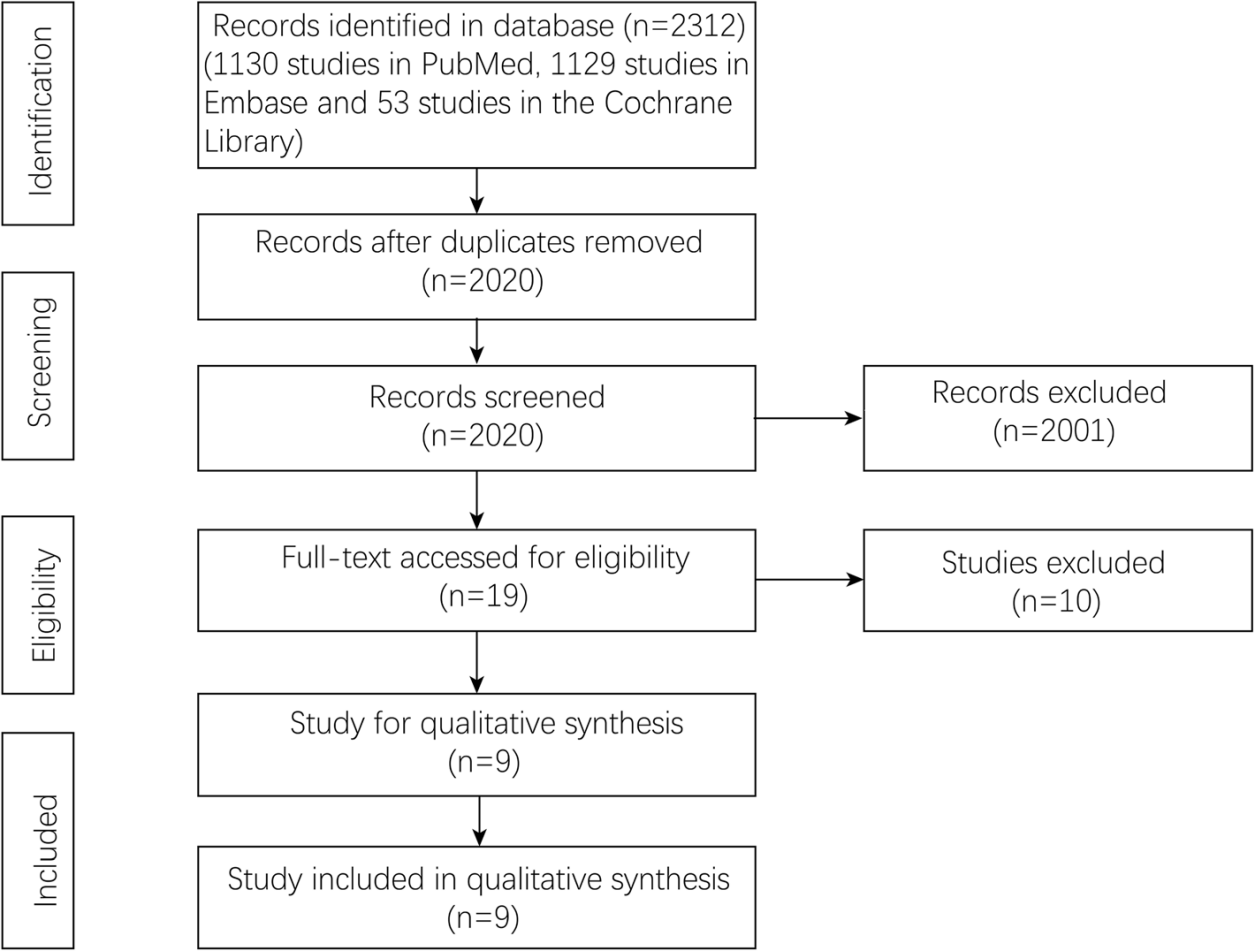
Flowchart of study selection

A total of 2312 studies (1130 studies in PubMed, 1129 studies in Embase, and 53 studies in the Cochrane Library) were screened in this meta-analysis. There were 2020 studies after removing the duplications. The title and abstract were screened by two authors independently, and 19 studies were evaluated at the full-text level. Case reports, reviews, letters, conferences, and comments were excluded. Finally, 9 studies [13–16, 22–26] that compared an effect of ER-LG group and LG-only group after laparoscopic surgery for gastric cancer were included in this meta-analysis (Fig. 1).

### Patient characteristics and quality assessment of the included studies

There were 595 patients who underwent laparoscopic gastrectomy after ER and 3016 patients who only underwent laparoscopic gastrectomy in the 9 studies. The publication year of the 9 studies were from 2012 to 2020, and all of the studies were retrospective studies. Seven studies were from Japan, and 2 studies were from Korea. The grade of complications and the scores of the Newcastle-Ottawa Scale of each study are shown in Table 1.

**Table 1:** Characteristics of the studies included in the meta-analysis

Author	Year published	Country	Study design	Study date	Sample size		Postoperative complications Clavien-Dindo classification (I/II/III/IV/V)		NOS
					ER-LG group	LG-only group	ER-LG group	LG-only group	
Ebihara Y	2015	Japan	Retrospective	2006-2013	38	38	II/III	II/III	8
Lee EG	2017	Korea	Retrospective	2003-2013	199	1505	I/II/III/IV/V	I/II/III/IV/V	8
Lee H	2019	Korea	Retrospective	2013-2018	107	428	Unknown	Unknown	8
Tsujimoto H	2012	Japan	Retrospective	2008-2010	17	80	Unknown	Unknown	7
Shindo K	2020	Japan	Retrospective	2000-2014	47	94	III/IV/V	III/IV/V	7
Komatsu S	2013	Japan	Retrospective	2007-2011	30	172	Unknown	Unknown	7
Aoyama J	2020	Japan	Retrospective	2013-2018	21	21	II/III/IV/V	II/III/IV/V	8
Jiang X	2012	Japan	Retrospective	2006-2009	111	600	Unknown	Unknown	8
Suzuki T	2013	Japan	Retrospective	2000-2010	25	78	Unknown	Unknown	7

Abbreviations: *ER* endoscopic resection, *LG* laparoscopic gastrectomy, *NOS* Newcastle-Ottawa Scale

### Baseline information

Baseline information, including age, sex, body mass index (BMI), tumor size, comorbidity, tumor depth, American Society of Anesthesiologists (ASA) score, and degree of pathological differentiation was extracted. After pooling all of the data, the ER-LG group had a younger age (MD=4.08, 95% CI=2.10 to 6.07, *P*<0.0001), more males (OR=1.51, 95% CI=1.23 to 1.85, *P*<0.0001), lower comorbidity (OR=1.76, 95% CI=1.23 to 2.51, *P*=0.002), a smaller tumor size (MD=−0.77, 95% CI=−1.00 to −0.55, *P*<0.00001) and a lower T stage (T1-T2: OR=2.42, 95% CI=1.09 to 5.34, *P*=0.03; T3-T4: OR=0.41, 95% CI=0.19 to 0.91, *P*=0.03) than the LG-only group. However, no difference was found in terms of BMI (MD=0.08, 95% CI=−0.21 to 0.37, *P*=0.58), ASA score (ASA1: OR=1.00, 95% CI=0.56 to 1.77, *P*=1.00; ASA2: OR=1.03, 95% CI=0.59 to 1.80, *P*=0.92) or degree of pathological differentiation (undifferentiated: OR=0.28, 95% CI=0.07 to 1.18, *P*=0.08; differentiated: OR=3.52, 95% CI=0.85 to 14.60, *P*=0.08) (Table 2).

**Table 2:** Summary of characteristics between ER-LG group and LG-only group

Characteristics	Studies	Participants (ER-LG/LG-only)	Mean difference (95% CI)	Heterogeneity
Baseline information				
Age, year	7	540/2766	4.08 [2.10, 6.07]; *P*<0.0001	I^2^=71%; *P*=0.002
Male	9	595/3016	1.51 [1.23, 1.85]; *P*<0.0001	*I*^2^=39%; *P*=0.11
BMI, kg/m^2^	8	570/2938	0.08 [−0.21, 0.37]; *P*=0.58	*I*^2^=0%; *P*=0.50
Tumor size, cm	3	237/1606	−0.77 [−1.00, −0.55]; *P*<0.00001	*I*^2^=0%; *P*=0.63
ASA 1	3	106/153	1.00 [0.56, 1.77]; *P*=1.00	*I*^2^=0%; *P*=0.81
ASA 2	3	106/153	1.03 [0.59,1.80]; *P*=0.92	*I*^2^=0%; *P*=0.87
T1-T2	7	463/2510	2.42 [1.09, 5.34]; *P*=0.03	*I*^2^=40%; *P*=0.13
T3-T4	7	463/2510	0.41 [0.19, 0.91]; *P*=0.03	*I*^2^=40%; *P*=0.13
Pathological undifferentiated	3	89/231	0.28 [0.07, 1.18]; *P*=0.08	*I*^2^=70%; *P*=0.04
Pathological differentiated	3	89/231	3.52 [0.85, 14.60]; *P*=0.08	*I*^2^=70%; *P*=0.04
Comorbidity	3	166/718	1.76 [1.23, 2.51]; *P*=0.002	*I*^2^=21%; *P*=0.28
Surgical methods and reconstruction methods				
Total gastrectomy	4	275/1644	0.94 [0.59, 1.49]; *P*=0.79	*I*^2^=0%; *P*=0.86
Subtotal gastrectomy	4	275/1644	1.07 [0.70, 1.65]; *P*=0.75	*I*^2^=0%; *P*=0.86
B-I or B-II	4	290/1200	1.09 [0.61, 1.93]; *P*=0.77	*I*^2^=62%; *P*=0.05
Roux-en-Y	4	290/1200	0.93 [0.64, 1.34]; *P*=0.69	*I*^2^=21%; *P*=0.28
Short-term outcomes				
Intraoperative blood loss	6	442/2489	−1.42 [−22.00, 19.16]; *P*=0.89	*I*^2^=77%; *P*=0.0005
Time to first oral feeding	3	55/118	−0.02 [−0.34, 0.31]; *P*=0.93	*I*^2^=0%; *P*=0.45
Retrieved lymph nodes	6	350/1412	−2.62 [−5.32, 0.07]; *P*=0.06	*I*^2^=72%; *P*=0.003
Metastatic lymph nodes	7	459/2338	0.66 [0.43, 1.00]; *P*=0.05	*I*^2^=0%; *P*=0.50
Postoperative hospital stay	6	442/2489	0.54 [−0.27, 1.35]; *P*=0.19	*I*^2^=0%; *P*=1.00
Conversion to open surgery	3	357/2199	0.73 [0.29, 1.86]; *P*=0.51	*I*^2^=0%; *P*=0.73
Postoperative complications				
Grade ≥ II	4	305/1658	1.11 [0.71, 1.73]; *P*=0.64	*I*^2^=0%; *P*=0.69
Grade ≥ III b	3	284/1637	1.47 [0.49, 4.43]; *P*=0.49	*I*^2^=0%; *P*=0.50
Anastomotic leakage	7	544/2832	1.20 [0.55, 2.65]; *P*=0.65	*I*^2^=0%; *P*=0.79
Anastomotic stenosis	4	369/2049	1.34 [0.54, 3.33]; *P*=0.53	*I*^2^=0%; *P*=0.66
Intestinal obstruction	7	522/2807	1.70 [0.93, 3.13]; *P*=0.08	*I*^2^=10%; *P*=0.36
Abdominal infection	5	420/2315	1.59 [0.71, 3.56]; *P*=0.26	*I*^2^=0%; *P*=0.65
Gastric stasis	5	472/2651	0.71 [0.31, 1.65]; *P*=0.43	*I*^2^=0%; *P*=0.93
Wound infection	6	519/2745	1.68 [0.84, 3.35]; *P*=0.14	*I*^2^=0%; *P*=0.90
Pancreatic leakage	5	328/1238	1.25 [0.53, 2.94]; *P*=0.62	*I*^2^=9%; *P*=0.36
Post-operative bleeding	4	464/2627	0.69 [0.24, 2.01]; *P*=0.50	*I*^2^=0%; *P*=1.00
Short-term death	3	353/2027	2.65 [0.77, 9.09]; *P*=0.12	*I*^2^=0%; *P*=0.88

Abbreviations: *ER* endoscopic resection, *LG* laparoscopic gastrectomy, *ASA* American Society of Anesthesiologists, postoperative complications (grade ≥ II and grade ≥ III b) were graded by the Clavien-Dindo classification

### Surgical methods and reconstruction methods

The surgical methods were divided into total gastrectomy and subtotal gastrectomy, and no difference was found between the ER-LG group and the LG-only group (total gastrectomy: OR=0.94, 95% CI=0.59 to 1.49, *P*=0.79; subtotal gastrectomy: OR=1.07, 95% CI=0.70 to 1.65, *P*=0.75). Similarly, reconstruction methods were found no statistical significance in both groups as well (B-I or B-II: OR=1.09, 95% CI=0.61 to 1.93, *P*=0.77; Roux-en-Y: OR=0.93, 95% CI=0.64 to 1.34, *P*=0.69) (Table 2).

### Operation time

**Fig. 2:**
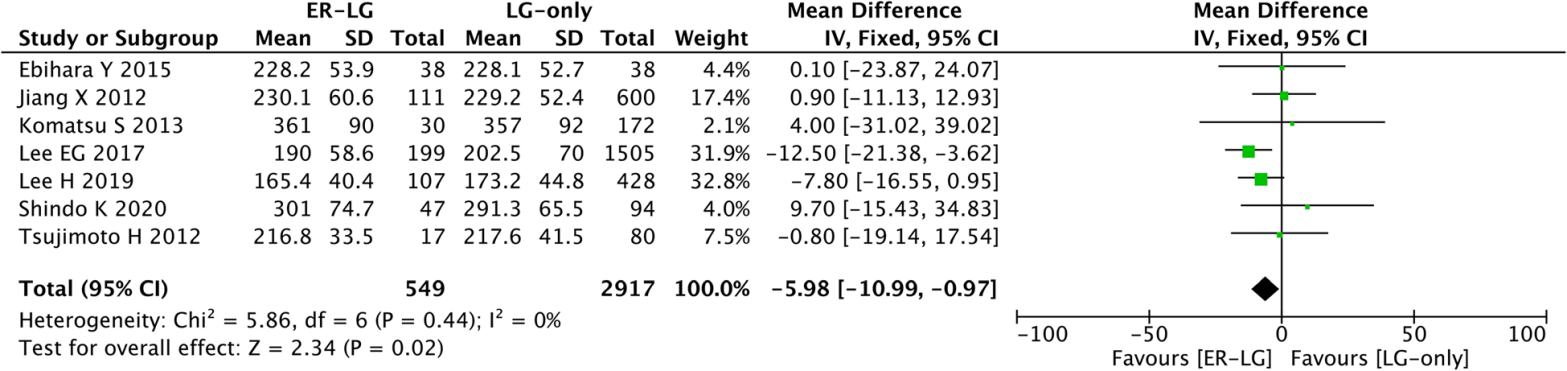
Forest plot of the operation time. 95% CI, 95% confidence interval; ER, endoscopic resection; LG, laparoscopic gastrectomy

Seven studies reported the operation time for laparoscopic gastrectomy, and the ER-LG group showed shorter operation time than the LG-only group (MD=−5.98, 95% CI=−10.99 to −0.97, *P*=0.02) (Fig. 2).

### Short-term surgical outcomes

Intraoperative blood loss, time to first oral feeding, retrieved lymph nodes, metastatic lymph nodes, postoperative hospital stay, and conversion to open surgery were extracted. No significance was found between the ER-LG group and the LG-only group in terms of intraoperative blood loss (MD=−1.42, 95% CI=−22.00 to 19.16, *P*=0.89), time to first oral feeding (MD=−0.02, 95% CI=−0.34 to 0.31, *P*=0.93), retrieved lymph nodes (MD=−2.62, 95% CI=−5.32 to 0.07, *P*=0.06), metastatic lymph nodes (MD=0.66, 95% CI=0.43 to 1.00, *P*=0.05), postoperative hospital stay (MD=0.54, 95% CI=−0.27 to 1.35, *P*=0.19) or conversion to open surgery (OR=0.73, 95% CI=0.29 to 1.86, *P*=0.51) (Table 2).

### Postoperative complications

**Fig. 3:**
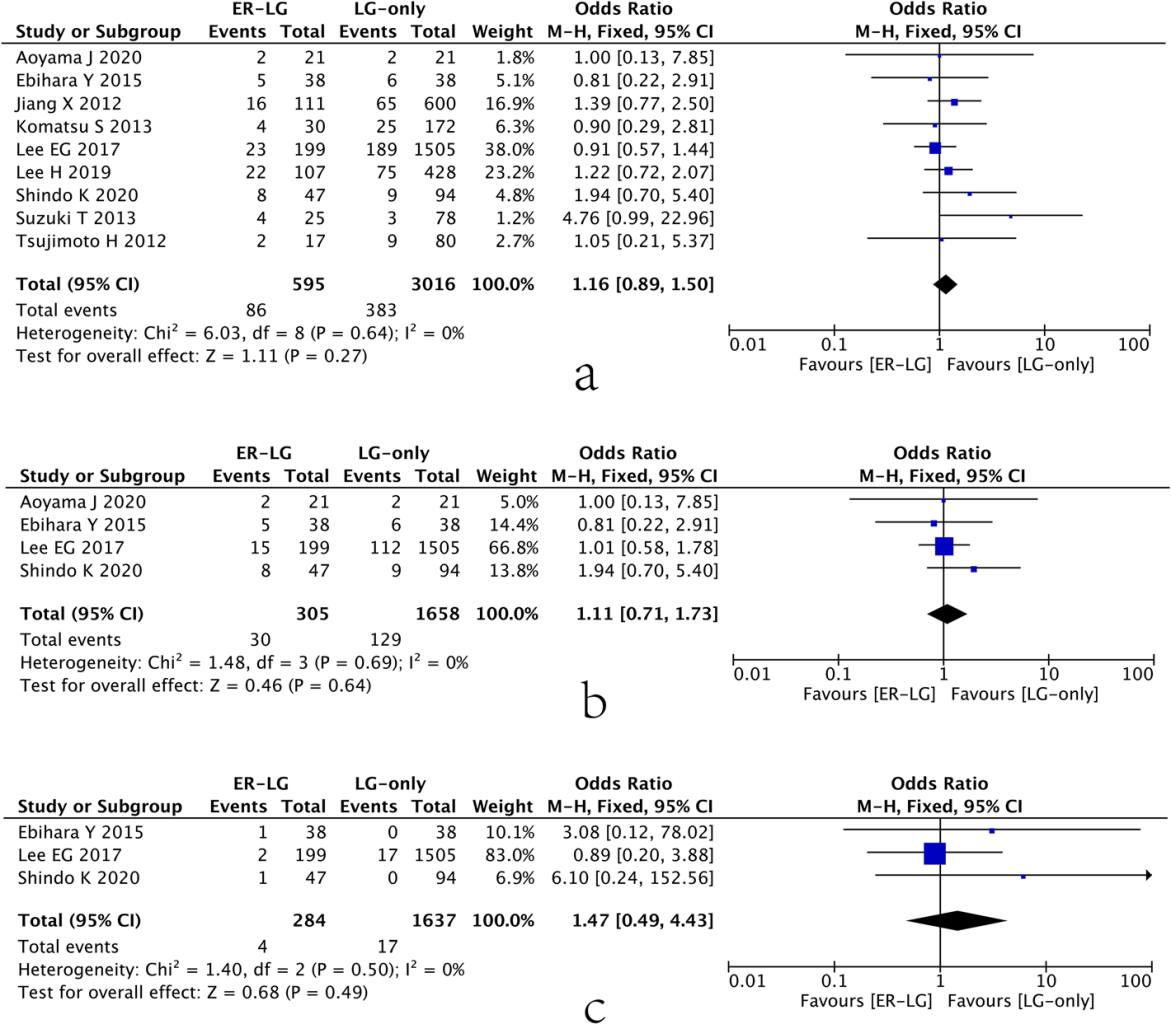
Forest plot showing postoperative complications. (**a**) Overall postoperative complications; (**b**) complications of grade ≥ II; (**c**) complications of grade ≥ III b. 95% CI, 95% confidence interval; ER, endoscopic resection; LG, laparoscopic gastrectomy

Complications after laparoscopic gastrectomy were extracted from 9 studies. After pooling all of the data, no significance was found between the ER-LG group and the LG-only group (OR=1.16, 95% CI=0.89 to 1.50, *P*=0.27) (Fig. 3a). According to the Clavien-Dindo classification, grade ≥ II and grade ≥ III b were extracted separately for analysis, and no difference was found in grade ≥ II (OR=1.11, 95% CI=0.71 to 1.73, *P*=0.64) or grade ≥ III b (OR=1.47, 95% CI=0.49 to 4.43, *P*=0.49) between the ER-LG group and the LG-only group (Fig. 3b and c). Moreover, specific complications including anastomotic leakage, anastomotic stenosis, intestinal obstruction, abdominal infection, gastric stasis, wound infection, pancreatic leakage, post-operative bleeding, and short-term death were found no statistical significance between the ER-LG group and the LG-only group (Table 2).

### Subgroup analysis of propensity score matching and non-propensity score matching studies

The subgroup analysis was performed between propensity score matching (PS) and non-propensity score matching (non-PS) studies. In baseline information of non-PS studies, the ER-LG group had more males (OR=1.83, 95% CI=1.43 to 2.34, *P*<0.01) and younger age (MD=6.21, 95% CI=4.24 to 8.19, *P*<0.01) than the LG-only studies. And in baseline information of PS studies, the ER-LG group had younger age (MD=2.66, 95% CI=0.96 to 4.36, *P*=0.002) than the LG-only group. In terms of short-term outcomes of non-PS studies, the ER-LG group had less retrieved lymph nodes (MD=−3.14, 95% CI=−5.01 to −1.27, *P*=0.001) and less metastatic lymph nodes (MD=0.52, 95% CI=0.30 to 0.92, *P*=0.02) than the LG-only group. No difference was found in operation time or complications of the PS studies (Table 3).

**Table 3:** Summary of characteristics in PS and non-PS studies

Characteristics	PS/non-PS		
	Studies	Mean difference (95% CI)	Heterogeneity
Baseline information			
Male	4/5	0.94 [0.65,1.35]; *P*=0.73/1.83 [1.43, 2.34]; *P*<0.01	*I*^2^=0%; *P*=0.91/*I*^2^=13%; *P*=0.33
Age	4/3	2.66 [0.96, 4.36]; *P*=0.002/6.21 [4.24, 8.19]; *P*<0.01	*I*^2^=25%; *P*=0.26/*I*^2^=57%; *P*=0.10
BMI	4/4	−0.13 [−0.60, 0.34]; *P*=0.58/0.21 [−0.16, 0.58]; *P*=0.26	*I*^2^=0%; *P*=0.98/*I*^2^=38%; *P*=0.18
T1-T2	3/4	2.04 [0.40, 10.28]; *P*=0.39/1.79 [0.30, 10.74]; *P*=0.53	*I*^2^=0%; *P*=0.49/*I*^2^=65%; *P*=0.04
T3-T4	3/4	0.49 [0.10, 2.48]; *P*=0.39/0.56 [0.09, 3.37]; *P*=0.53	*I*^2^=0%; *P*=0.49/*I*^2^=65%; *P*=0.04
Surgical methods and reconstruction methods			
TG	2/2	1.56 [0.42, 5.88]; *P*=0.51/0.87 [0.53, 1.44]; *P*=0.60	*I*^2^=0%; *P*=0.98/*I*^2^=0%; *P*=0.72
SG	2/2	0.64 [0.17, 2.41]; *P*=0.51/1.14 [0.72, 1.80]; *P*=0.58	*I*^2^=0%; *P*=0.98/*I*^2^=0%; *P*=0.73
B-I or B-II	2/2	0.91 [0.55, 1.52]; *P*=0.72/1.13[0.31, 4.09]; *P*=0.85	*I*^2^=0%; *P*=0.84/*I*^2^=77%; *P*=0.04
Roux-en-Y	2/2	1.10 [0.66, 1.83]; *P*=0.72/0.96 [0.30, 3.04]; *P*=0.94	*I*^2^=0%; *P*=0.84/*I*^2^=67%; *P*=0.08
Short-term outcomes			
Blood loss	2/4	12.97 [−60.39, 86.34]; *P*=0.73/−6.44 [−17.08, 4.20]; *P*=0.24	*I*^2^=91%; *P*=0.0007/*I*^2^=11%; *P*=0.34
Operation time	3/4	−5.27 [−13.08, 2.55]; *P*=0.19/−6.48[−13.02, 0.06]; *P*=0.05	*I*^2^=0%; *P*=0.39/*I*^2^=24%; *P*=0.27
Retrieved LNs	3/3	−2.33 [−7.87, 3.21]; *P*=0.41/−3.14 [−5.01, −1.27]; *P*=0.001	*I*^2^=82%; *P*=0.004/*I*^2^=0%; *P*=0.70
Metastatic LNs	4/3	0.96 [0.50, 1.81]; *P*=0.89/0.52 [0.30, 0.92]; *P*=0.02	*I*^2^=0%; *P*=0.62/*I*^2^=12%; *P*=0.32
PHS	2/4	0.60 [−1.25,2.45]; *P*=0.52/0.53 [−0.37, 1.43]; *P*=0.25	*I*^2^=0%; *P*=1.00/*I*^2^=0%; *P*=0.95
Postoperative complications			
Overall	4/5	1.25 [0.81, 1.92]; *P*=0.32/1.11[0.80, 1.54]; *P*=0.53	*I*^2^=0%; *P*=0.75/*I*^2^=15%; *P*=0.32
AL	3/4	1.18 [0.22, 6.28]; *P*=0.84/1.21 [0.49, 2.97]; *P*=0.68	*I*^2^=0%; *P*=0.77/*I*^2^=0%; *P*=0.45
AS	2/2	2.27 [0.59, 8.74]; *P*=0.24/0.86 [0.23, 3.28]; *P*=0.83	*I*^2^=0%; *P*=0.49/*I*^2^=0%; *P*=0.92
Ileus	2/5	1.90 [0.45, 8.01]; *P*=0.38/ 1.66 [0.85, 3.25]; *P*=0.14	*I*^2^=0%; *P*=0.72/*I*^2^=39%; *P*=0.16
Abdominal infection	2/3	0.46 [0.05, 4.47]; *P*=0.50/2.06 [0.87, 4.87]; *P*=0.10	*I*^2^=0%; *P*=0.76/*I*^2^=0%; *P*=0.61
Gastric stasis	2/3	0.60 [0.15, 2.36]; *P*=0.47/0.79 [0.27, 2.28]; *P*=0.66	*I*^2^=0%; *P*=0.38/*I*^2^=0%; *P*=0.98
Wound infection	3/3	2.31 [0.78, 6.86]; *P*=0.13/1.33[0.53, 3.36]; *P*=0.55	*I*^2^=0%; *P*=0.65/*I*^2^=0%; *P*=0.96
PL	3/2	2.17 [0.59, 7.97]; *P*=0.24/0.80 [0.23, 2.77]; *P*=0.72	*I*^2^=0%; *P*=0.37/*I*^2^=9%; *P*=0.29
PB	2/2	0.71 [0.18, 2.81]; *P*=0.63/0.67 [0.12, 3.59]; *P*=0.64	*I*^2^=0%; *P*=0.96/*I*^2^=0%; *P*=0.91

Abbreviations: *ER* endoscopic resection, *LG* laparoscopic gastrectomy, *PS* propensity score matching, *TG* total gastrectomy, *SG* subtotal gastrectomy, *LNs* lymph nodes, *PHS* postoperative hospital stay, *AL* anastomosis leakage, *AS* anastomotic stenosis, *PL* pancreatic leakage, *PB* postoperative bleeding

### Sensitivity, consistency, *I*^2^, and publication bias

**Fig. 4:**
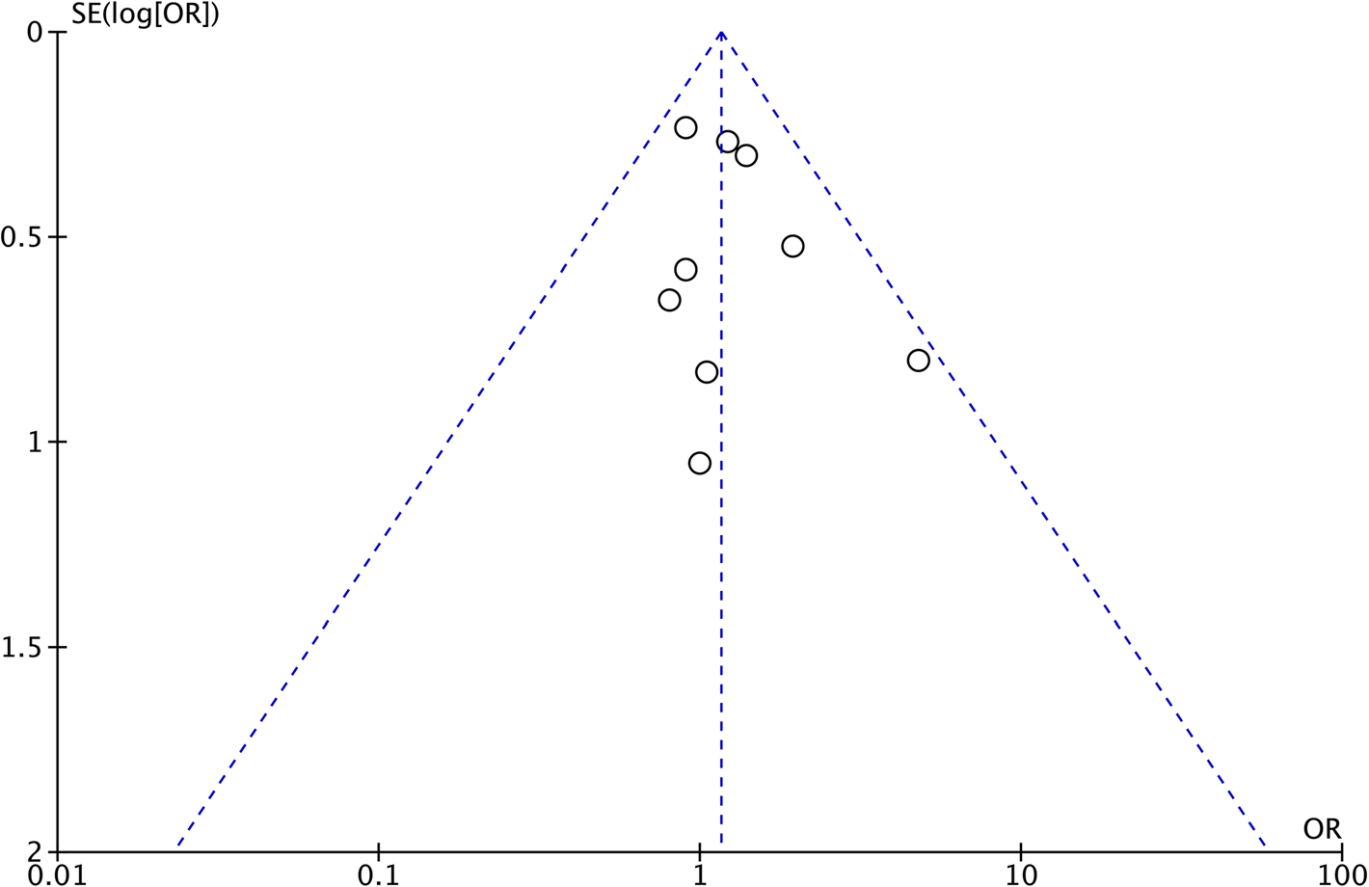
Funnel plot of overall postoperative complications. SE, standard error; OR, odds ratio

Repeated meta-analyses were performed by excluding one study in turn as a sensitivity analysis was performed to evaluate the impact of each individual study on the pooled OR or MD, and the results were found to be the same. Consistency was measured by estimating the degree of inconsistency among the results of the studies. Publication bias for the included studies was based on a visual inspection of the funnel plots, which were symmetrical, and no obvious publication bias was found (Fig. 4).

## Discussion

Nine studies were included in this meta-analysis. Tumor T stage, surgical methods, reconstruction methods, intraoperative blood loss, operation time, the total number of retrieved lymph nodes, the metastatic lymph nodes, conversion to open surgery, postoperative complications, and postoperative hospital stay were compared. Only the operation time was different, and the ER-LG group had a shorter operation time than the LG-only group. However, no difference was found in operation time after subgroup analysis of propensity score matching studies.

ER is a standard treatment for patients with early gastric cancer and a negligible risk of lymph node metastasis [27, 28]. Subsequent gastrectomy is required if the tumor is resected incompletely by ER. Tumor size (>3 cm), undifferentiated tumor type, and positive horizontal margins were predictors of residual incomplete gastrectomy [4]. A previous study found that LG contributed to the effectiveness of the treatment of choice for noncurative ER compared with open gastrectomy [26].

LG is currently widely accepted nowadays. Age, obesity, tumor stage, and ASA grade are potential risk factors that affect the short-term outcomes of LG [29–31]. However, whether a previous ER has an effect on the short-term outcomes of LG remains controversial.

Intraoperative blood loss, operation time, surgical methods, reconstruction methods, total number of retrieved lymph nodes, metastatic lymph nodes, conversion to open surgery, postoperative complications, and postoperative hospital stay were not significantly different between the ER-LG group and the LG-only group in this meta-analysis. The ER-LG group had a shorter operation time than the LG-only group, and these results were reported in previous studies [13]. The results of the operation time remained unclear. In this meta-analysis, we compared the baseline information between the ER-LG group and the LG-only group. ER-LG group had a smaller tumor size and a lower T stage than the LG-only group, which might contribute to the shorter operation time [13].

In addition to the short-term outcomes of the ER-LG group and LG-only group, the previous literature mentioned some other factors that affected the subsequent LG after ER. The ER procedure can cause ulcers in the stomach, and patients can suffer from inflammation, subsequent fibrosis, and even adhesion to the outer wall of the stomach. Ulcers caused by ER reached the healing or scarring stage within 4-8 weeks [32, 33]. Therefore, a shorter interval between ER and LG might cause a longer operation time and a larger amount of blood loss [23]. After ER, larger artificial ulcers (> 25 mm) and intra-abdominal adhesions are usually observed, which makes additional laparoscopic gastrectomy difficult [26, 34]. Perforation of the stomach might cause more adhesions and increase the difficulty of LG as well [14]. A previous study reported that ER affected the preservation of the celiac branch of the vagus nerve when undergoing LG, which meant that ER might have a negative impact on gastrointestinal motility [16].

The propensity score methods were performed to minimize discrepancy in clinical characteristics between the ER-LG group and LG-only group. Therefore, for more precise results, subgroup analysis of PS studies and non-PS studies were performed in this meta-analysis. However, no difference was found in operation time or complications after subgroup analysis of PS studies, and the consistent baseline information of PS studies might contribute to the results. Nevertheless, the sample size of PS studies was limited; thus, studies with PS in a large sample size should be performed in the future.

This meta-analysis had some limitations as well. First, only nine retrospective studies were involved without any cohort studies or randomized controlled trials. Second, the included studies were only from Japan and Korea; therefore, the current results applied to restricted areas. Large-scale of randomized controlled trials need to be carried out in the future. Third, the length of interval time between ER and LG was lacking, which might affect the short-term outcomes, and furthermore, the overall survival between the ER-LG group and LG-only group was not mentioned in any of the studies. Therefore, more details of ER-LG are needed in the future. In addition, the current study was a meta-analysis of retrospective studies with aggregate data (AD) rather than individual participant data (IPD). Various factors were involved in the decision of the procedure, and detailed indications for each gastrectomy option likely differ among facilities, which could be a potential source of bias. Finally, ER included ESD and EMR, and these two different methods might result in different outcomes between the ER-LG group and the LG-only group; however, previous studies did not clearly separate ESD and EMR, which needs to be done in future studies.

## Conclusion

In conclusion, ER did not affect subsequent LG in terms of short-term outcomes, and the ER-LG group might have a shorter operation time than the LG-only group.

## Data Availability

All data generated or analyzed during this study are included in this published article.

## Abbreviations

ER: Endoscopic resection
LG: Laparoscopic gastrectomy
ESD: Endoscopic submucosal dissection
EMR: Endoscopic mucosal resection
BMI: Body mass index
NOS: Newcastle-Ottawa Scale
SD: Standard deviations
ORs: Odds ratios
MDs: Mean differences
CI: Confidence intervals
ASA: American Society of Anesthesiologists
PS: Propensity score matching
AD: Aggregate data
IPD: Individual participant data
